# Validation of Emergency Surgery Score (ESS) as outcome prediction score in Egyptian patients undergoing emergency laparotomy

**DOI:** 10.1186/s12245-025-00934-z

**Published:** 2025-07-07

**Authors:** Mina Montasser, Doaa Ahmed Mohamed Ellisy, Jehan Ahmed Sayed, Momen Mostafa Makkey

**Affiliations:** 1 Emergency Medicine Department, Faculty of Medicine, Alexandria University, Alexandria, Egypt; 2 Department of Emergency, Faculty of medicine, Assiut University, Assiut, Egypt; 3 Anesthesia and Intensive Care Department, Faculty of Medicine, Assiut University, Assiut, Egypt

**Keywords:** The emergency surgery score, Emergency laparotomy complications, Postoperative mortality rate

## Abstract

**Background:**

Emergency laparotomy is an exploratory procedure for many surgical situations. Proper prognostic prediction helps recognize high-risk patients who benefit from further therapy. The Emergency Surgery Score (ESS) was developed as a preoperative risk evaluation assessment that predicts patient outcomes after emergency laparotomy.

**Objective:**

This study is to validate the ESS in Egyptian patients by evaluating its predictive ability for 30-day mortality, surgical complications, and ICU admission rates.

**Methods:**

This prospective observational cohort study was performed out from May 2022 to November 2023 at two tertiary centers in Egypt. Patients aged 18 and above undergoing emergency laparotomy were included, whereas pregnant women and individuals with vascular or gynecological indications were eliminated. Preoperative, intraoperative, and postoperative data were thoroughly collected, and ESS was calculated for all patients. The primary desired outcome was 30-day death, while secondary outcomes included surgical complications and ICU admission. Standard definitions from the American College of Surgeons National Surgical Quality Improvement Program (ACS-NSQIP) were used.

**Results:**

This study included a diverse cohort, with a mean age of the patients 48.66 ± 19.37 years. The 30-day totally mortality rate was 28%. The most common complication was pneumonia (60%), followed by sepsis and acute kidney injury. Higher ESS scores were significantly associated with increased mortality and complication rates, confirming its predictive validity.

**Conclusion:**

ESS effectively categorized Egyptian patients who more reliable to complications of emergency laparotomy. Its use may improve clinical decision-making, optimize resource allocation, and facilitate patient and family counseling. Further research is recommended to refine its applicability across different populations.

**Trial registration:**

NCT05639920 registration date on (26/10/2021).

## Introduction

Emergency laparotomy is a common and often life-saving surgical intervention in clinical practice. Each patient presents with a unique clinical picture, underlying pathology, surgical anatomical site, and prior treatment [1].

This surgical category encompasses a wide array of procedures, with over 400 OPCS codes used to classify the various types of emergency laparotomies, highlighting the heterogeneity of this patient population [2].

International reports indicate that the 30-day mortality rate following emergency laparotomy ranges from 13 to 18%, despite significant advancements in surgical techniques and postoperative supportive care [3].

The implementation of an effective scoring system can predict and identify high-risk patients, thereby improving clinical decision-making through the evaluation of potentially life-threatening consequences. The ESS stratifies patients into distinct risk categories, which aids in prognostication and the identification of individuals who may require critical care postoperatively. Furthermore, it assists in anticipating intraoperative challenges, enabling surgeons to determine the most appropriate surgical approach for each patient [4, 5].

The Emergency Surgery Score (ESS) was developed in 2016 as a preoperative risk assessment tool for forecasting patient outcomes, specifically 30-day mortality, surgical complications, and the need for intensive care unit (ICU) admission [6, 7].

The ESS has demonstrated its reliability as a predictor of outcomes in emergency laparotomy and may serve as a valuable bedside tool for clinical decision-making. It assists in counseling preoperative patients and their families while also facilitating appropriate risk adjustment in quality benchmarking for emergency laparotomies [8].

The ESS has been validated as an outcome prediction score in diverse populations through studies conducted in numerous countries, including the USA, the UK [9], Greece [10], Raffee et al. [11], and Saudi Arabia [12].

This study focused on the validation of the ESS score specifically within the Egyptian population, as its utility in this context has not yet been evaluated.

## Patients and methodologies

### Study design

A prospective observational cohort study was conducted. The research was carried out at two tertiary centers (the emergency unit at Assiut University Hospital and the emergency department at Alexandria University Hospital) from May 2022 to November 2023, following the approval of the Assiut University Faculty of Medicine’s Research Ethics Committee (IRB local approval number 17101900). The trial was registered on ClinicalTrials.gov under the identification number NCT05639920. Informed written consent was obtained from all patients or their first-degree family members prior to enrollment, after they were fully informed about the study.

### Inclusion criteria

Patients aged 18 years and older undergoing emergency laparotomy were included in the study. Emergency laparotomy was defined as any laparotomy performed immediately after confirming the diagnosis or the emergence of associated preoperative symptoms, where a prolonged delay could compromise the patient’s well-being and prognosis [13].

### Exclusion criteria

Pregnant women and individuals with vascular or gynecological indications for laparotomy were excluded to reduce heterogeneity, as these patient populations differ from the general population undergoing emergency laparotomy, which could confound the ESS validation. Patients unavailable for follow-up until the 30th postoperative day were also excluded.

### Sample size

The sample size was calculated using Epi-Info 7, based on a 30-day mortality rate after emergency laparotomy of 10% as reported in a previous study [14], with a confidence limit of 5% and a confidence level of 95%. The minimum number of patients required for this study was calculated to be 137.

### Patient recruitment

**Fig. 1:**
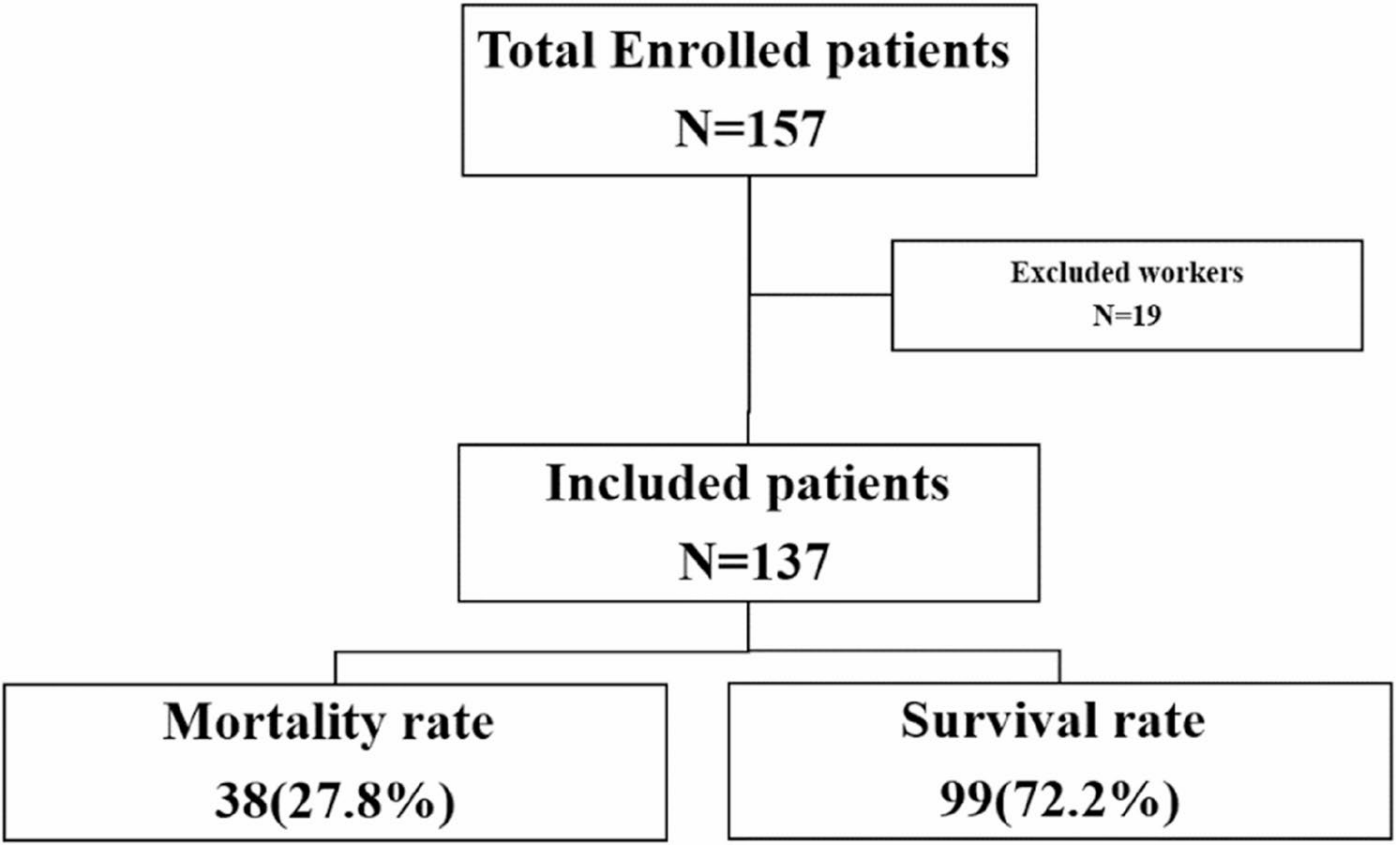
Flow chat of patient’s enrollment

Patients were recruited using a simple random sampling technique. A total of 137 patients were included in the final analysis after excluding 19 patients, as depicted in the flow chart (Fig. 1).

#### Data collection

Detailed preoperative, intraoperative, and postoperative data were meticulously collected from all patients undergoing emergency laparotomy during the study period. Standard definitions from the American College of Surgeons National Surgical Quality Improvement Program (ACS–NSQIP) were adhered to [15].

### Preoperative

Information regarding personal history, including age, sex, and self-reported race, was obtained from all participants. Routine preoperative evaluation and optimization for emergency laparotomy were conducted. The laboratory investigations performed included serum albumin level, prothrombin time-international normalized ratio (PT-INR), liver function tests with alkaline phosphatase (ALP), kidney function tests with electrolytes, and complete blood count. Following the completion of the workup, diagnostic confirmation, and resuscitation, the patient underwent an emergency laparotomy. The surgical team, comprising resident doctors and consultants under the supervision of a professor from the general surgery department, was responsible for the operation.

The Emergency Surgery Score (ESS) was calculated prior to the surgical intervention.

### Emergency Surgery Score (ESS) calculation

The ESS for each patient was computed using the methodology outlined by Sangji et al. [15] and Sangji et al. [16], utilizing preoperative data elements defined by ACS-NSQIP. The ESS incorporates 22 independent predictors of mortality in patients undergoing emergency surgery, encompassing three demographic traits, ten comorbidities, and nine preoperative laboratory indicators. The total score ranges from 0 to 29. As all patients in this study identified as non-white, a value of ‘0’ was assigned for the corresponding data point. Consistent with the methodology employed by Naar et al. [17], any absent factors were assigned the default value, indicating they did not contribute to an increase in the final ESS score [17].

### Postoperative

Postoperatively, patients underwent clinical monitoring with laboratory and radiological assessments as clinically indicated until discharge. This was followed by weekly physical follow-up evaluations in the outpatient clinic, ward, or ICU for a period of 30 days.

The predictive ability of the ESS for 30-day mortality, surgical complications, and the need for intensive care unit admission was assessed.

### Defining outcomes

The primary endpoint of this study was 30-day postoperative mortality. The aim was to evaluate the ESS’s capacity to predict mortality during the initial hospitalization period. Additional secondary outcomes analyzed included ICU admission and readmission, and the duration of hospital stay. The primary endpoint of the current investigation was the prediction of 30-day mortality. Secondary outcomes included the incidence of at least one complication, the reoperation rate, and the rate of postoperative ICU readmission. Complications were defined according to ACS-NSQIP criteria and included superficial and deep surgical site infections, sepsis, septic shock, acute kidney injury, acute renal failure, pneumonia, respiratory failure, urinary tract infection (UTI), acute myocardial infarction, congestive heart failure, wound dehiscence, bleeding requiring transfusion or intervention, strokes or cerebrovascular events, unscheduled intubation, and cardiac arrest requiring cardiopulmonary resuscitation (CPR) [12].

### Statistics

Statistical analysis was performed using the Statistical Package for the Social Sciences (SPSS) version 27.0 (SPSS Inc., Chicago, IL, USA). The normality of data distribution was assessed using the Shapiro-Wilk and Kolmogorov-Smirnov tests. Descriptive statistics for quantitative data included the mean and standard deviation, while categorical data were summarized using frequencies. For categorical variables, significance testing was conducted using chi-square (χ²); for quantitative variables, independent samples t-tests and correlation analyses were employed. Receiver operating characteristic (ROC) curve analysis was utilized to determine the optimal cutoff value for the ESS, along with its corresponding sensitivity, specificity, positive predictive value (PPV), negative predictive value (NPV), and accuracy.

## Results

The mean age of the study population was 48.66 ± 19.37 years. The majority of the sample (57.7%) was under the age of 60, while 42.3% were 60 years or older. The sample exhibited a near-even distribution between males (53.3%) and females (46.7%). The mean time from diagnosis to surgical intervention was 6.54 ± 3.23 h.

A small proportion of the patients (12.4%) were transferred from an outside emergency department. Over one-fourth of the patients (27%) were transferred from an acute care hospital (Table 2).

Regarding comorbidities, ascites was present in a high percentage of the population (81%). More than half of the patients (56.9%) had a body mass index (BMI) below 20 kg/m². A notable percentage of patients (20.4%) had disseminated cancer. Nearly half of the patients (46.7%) experienced dyspnea. One-third of the patients (32.8%) required assistance with daily activities, indicating functional dependence. A considerable portion of the patients (39.4%) had a history of chronic obstructive pulmonary disease (COPD). Over half of the patients (55.5%) had hypertension, and a similar proportion were on steroid therapy (58.4%). More than half required ventilator support (53.3%) for at least 48 h prior to surgery. A significant portion of the patients had experienced weight loss (19.7%) (Table 1).

**Table 1:** The parameters used in the calculation of ESS among studied group

Variables	Frequency (%)
Demographics	
Age > 60 years	58(42.3%)
White race	0(0.0%)
Transfer status	
Outside emergency department	17(12.4%)
Acute care hospital inpatient facility	37(27%)
Comorbidities	
Ascites	111(81%)
BMI < 20 kg/m^2^	78(56.9%)
Disseminated cancer	28(20.4%)
Dyspnea	64(46.7%)
Functional dependence	45(32.8%)
History of COPD	54(39.4%)
Hypertension	76(55.5%)
Steroid use	80(58.4%)
Ventilator requirement within 48 h preoperatively	73(53.3%)
Weight loss > 10% in the preceding 6 months	27(19.7%)

A significant proportion of patients (36.5%) had low serum albumin levels (< 3.0 U/L). The majority of patients (66.4%) had elevated alkaline phosphatase levels (> 129 U/L). Over half of the patients (54%) had elevated aspartate aminotransferase (SGOT) levels (> 40 U/L). Nearly half of the patients (48.2%) had elevated blood urea nitrogen (BUN) levels (> 40 mg/dL), and a similar proportion (47.4%) had elevated creatinine levels (> 1.2 mg/dL). The international normalized ratio (INR) was greater than 1.5 in approximately one-quarter (23.4%) of the patients. The platelet count was below 150 × 10³/µL in nearly one-quarter of the patients (22.6%). Serum sodium levels were greater than 145 mg/dL in about one-quarter of the patients (24.1%). Regarding white blood cell (WBC) count, patients were categorized into three groups: a significant portion (18.2%) had a WBC count > 25 × 10³/µL, half of the patients (50.4%) had a WBC count between 15 and 25 × 10³/µL, while a small percentage (8%) had a low WBC count < 4.5 × 10³/µL (Table 2).

**Table 2:** The laboratory parameters used in the calculation of ESS among studied group

Laboratory values (%)	
Albumin < 3.0 U/L	50(36.5%)
Alkaline phosphatase < 3.0 U/L	91(66.4%)
Blood urea nitrogen > 40 mg/dL	66(48.2%)
Creatinine > 1.2 mg/dL	65(47.4%)
International normalized ratio > 1.5	32(23.4%)
Platelets < 150 × 103/µL	31(22.6%)
SGOT > 40 U/L	74(54%)
Sodium > 145 mg/dL	33(24.1%)
WBC	
WBC < 4.5 10^3^/µL	11(8%)
WBC > 15 and ≤ 25 10^3^/µL	69(50.4%)
WBC > 25 10^3^/µL	25(18.2%)

The majority of patients survived (72.2%), and the overall mortality rate was nearly 28%.

Pneumonia was the most common postoperative complication, affecting nearly 60% of patients. Over half of the patients (52.6%) experienced superficial surgical site infections. Almost half of the patients (44.5%) developed acute renal failure, and 43.1% of patients required inotropic drugs postoperatively. Urinary tract infections affected a significant proportion of patients (40.1%), and sepsis occurred in over one-third of the patients (37.2%) (Table 3).

**Table 3:** Incidence of postoperative complications among studied patients

Complications	Incidence (%)
Pneumonia	82(59.9%)
Superficial surgical site infection	72(52.6%)
Acute renal failure	61(44.5%)
Postoperative inotropic drug	59(43.1%)
ICU admission	58(42.3%)
Urinary tract infection	55(40.1%)
Sepsis	51(37.2%)
Renal insufficiency	50(36.5%)
Wound dehiscence	46(33.6%)
Bleeding requiring transfusion	41(29.9%)
Deep incisional surgical site infection	38(27.7%)
Ventilator requirement > 48 h	37(27%)
Septic shock	37(27%)
Unplanned intubation	35(25.5%)
Deep vein thrombosis/thrombophlebitis	35(25.5%)
Cardiac arrest requiring CPR	33(24.1%)
Myocardial infarction	31(22.6%)
Organ/space surgical site infection	29(21.2%)
Anastomosis leak	27(19.7%)
Pulmonary embolism	26(19%)
Stroke/cerebrovascular accident	22(16.1%)
Graft/prosthesis/flab failure	22(16.1%)

**Fig. 2:**
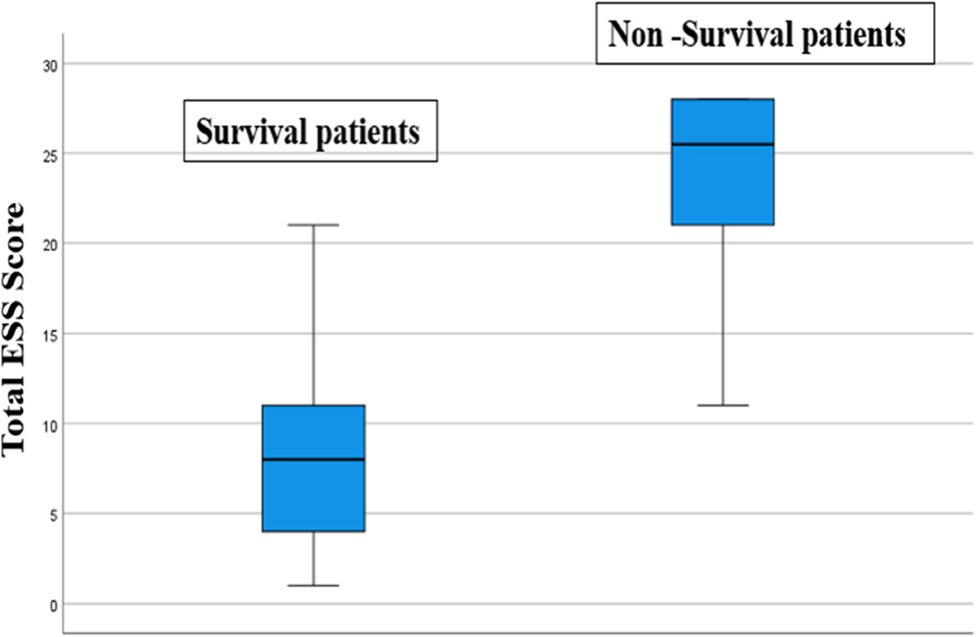
Comparison of total Ess score between survival and non-survival patients

The ESS total score was statistically significantly lower in surviving patients compared to non-surviving patients (*p* < 0.001) (Fig. 2).

**Fig. 3:**
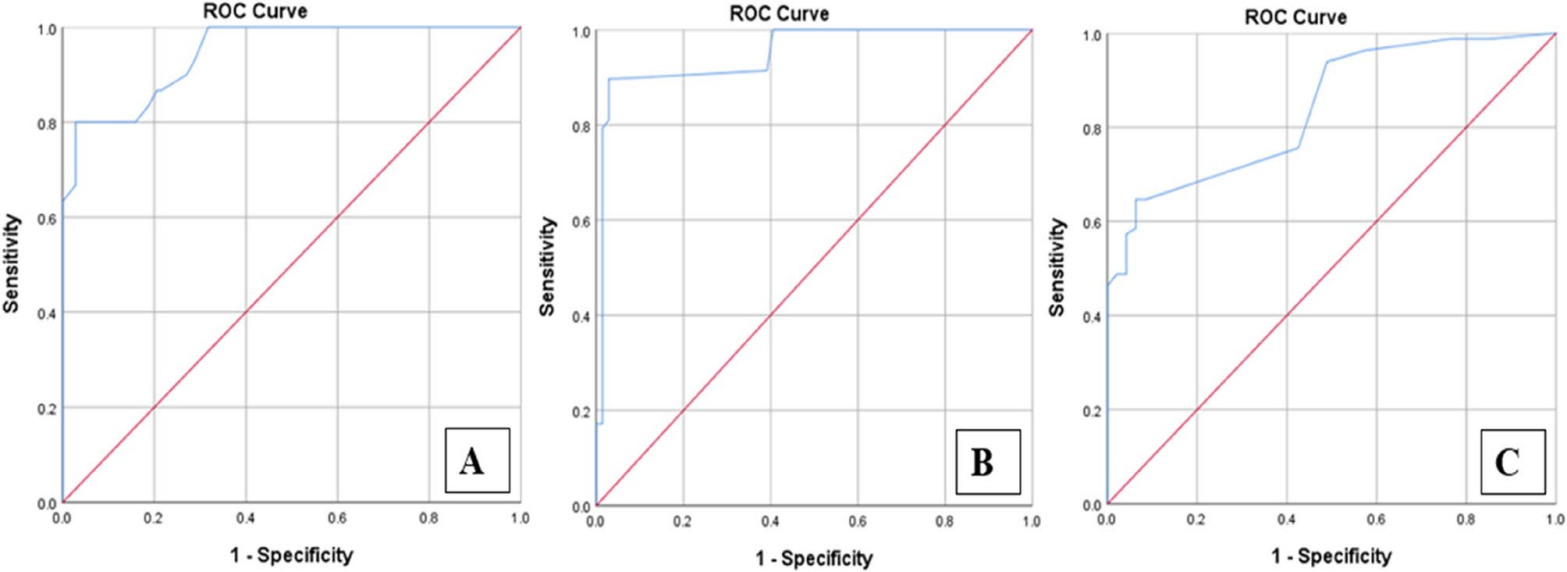
ROC Curve for the ESS versus 30-day mortality, ICU admission, and postoperative complications

The Area Under the Curve (AUC) for the ESS in predicting mortality was 0.946, indicating excellent predictive ability. A cutoff score of 12.00 provided the optimal balance between sensitivity (93.3%) and specificity (71%) for predicting mortality (Table 4, Fig. 3A).

**Table 4:** ROC curve for emergency surgery score to predicted mortality, ICU admission, and post operative complication

Item	AUC	Cutoff	Sensitivity	Specificity	*p*-value
Mortality	0.946	12.00	93.3%	71%	< 0.0001
ICU admission	0.950	7.50	91.4%	60.9%	< 0.0001
Post operative complication	0.839	6.00	81.7%	55.3%	< 0.0001

Regarding ICU admission, the AUC was 0.950, demonstrating that the ESS was effective at predicting the need for ICU admission. At a cutoff score of 7.5, the sensitivity and specificity were 91.4% and 60.9%, respectively (Fig. 3B).

Regarding postoperative complications, the AUC was 0.839, which is considered good, suggesting that the ESS had a strong predictive ability for postoperative complications. At a cutoff score of 6, the sensitivity and specificity were 81.7% and 55.3%, respectively (Fig. 2C).

Multivariate analysis identified several independent predictors of 30-day mortality following emergency laparotomy. These included advanced age (OR = 1.676, *p* = 0.001), higher Emergency Surgery Score (ESS) (OR = 3.979, *p* < 0.001), presence of preoperative ascites (OR = 2.418, *p* = 0.012), postoperative pneumonia (OR = 3.699, *p* = 0.006), and wound infection (OR = 2.765, *p* = 0.018). These findings suggest that both preoperative and postoperative factors contribute significantly to mortality risk, with the ESS serving as a robust predictor. In contrast, sex was not a significant predictor (*p* = 0.685) (Table 5).

**Table 5:** Multivariate logistic regression of mortality predictors among studied participant

Variables	Odd ratio	Lower level	Upper level	*p*-value
Age	1.676	1.226	2.291	0.001
Sex	1.055	0.815	1.366	0.685
Total score	3.979	1.516	5.539	< 0.001
Wound infection	2.765	1.597	15.795	0.018
Post operative pneumonia	3.699	2.876	5.682	0.006
Pre-operative ascites	2.418	1.218	4.798	0.012

## Discussion

In addition to mortality, the ESS predicts postoperative complications and the requirement for admission to the intensive care unit (ICU) [10].

This study is a prospective cohort study. All patients who met the inclusion criteria and underwent emergency laparotomies in the emergency departments of Alexandria University Hospital and Assiut University Hospital were included in the research. The average age of the 137 patients in the research was 48.66 ± 19.37 years, with 53.3% of the sample being male and 46.7% being female. The majority of the sample (57.7%) was under 60 years of age. In contrast to these findings, a multicenter prospective observational study utilizing the American College of Surgeons National Surgical Quality Improvement Program database from 2007 to 2017 reported a mean age of 60.5 years and a female representation of 50.3% among the participants [18].

This study demonstrated that a small proportion of the patients (12.4%) were transferred from an outside emergency department, while over one-fourth of patients (27%) were transferred from an acute care hospital.

A retrospective study conducted at a tertiary hospital in Jordan, which examined the predictive capacity and reliability of the ESS in the Jordanian surgical population, reported that 91.9% of patients were admitted through their emergency room, 6.8% were transferred from other hospitals, and 1.3% (17 patients) were moved from other inpatient departments to the surgery ward [11].

Regarding comorbidities, the results of this study indicated that more than half of the patients (56.9%) had a BMI below 20 kg/m², and over half of the patients (55.5%) had hypertension. A notable percentage of the patients (20.4%) had disseminated cancer.

A retrospective study at a Saudi academic health facility found that approximately 10% of patients had a BMI below 20 kg/m². The findings also showed that 6% of patients had cancer, and approximately 18% of their study population had diabetes and hypertension [12].

The present study results reported that more than half of the patients were on steroids and required ventilator support for at least 48 h before surgery (58.4% and 53.3%, respectively). One-third of the patients (32.8%) required assistance with daily activities, indicating functional dependence.

These findings differed from a prospective observational cohort study conducted in two major academic facilities in Thessaloniki, Greece, and Boston, Massachusetts, USA, which reported much lower rates of functional dependence (10%) among Greek patients. Moreover, the prevalence of steroid use was minimal (1%), and the incidence of preoperative ventilator dependence was also reduced (2%) in the Greek cohort [10].

This study found that a significant portion of patients (36.5%) had low albumin levels (< 3.0 U/L). The majority of patients (66.4%) had high alkaline phosphatase levels (> 129 U/L). Over half of the patients (54%) had high SGOT levels (> 40 U/L). Nearly half of the patients (48.2%) had high BUN levels (> 40 mg/dL), and a similar proportion (47.4%) had high creatinine levels (> 1.2 mg/dL). The INR was greater than 1.5 in about a quarter (23.4%) of the patients. The platelet count was lower than 150 × 10³/µL in nearly a quarter of the patients (22.6%). Serum sodium levels were greater than 145 mg/dL in about a quarter of the patients (24.1%). Regarding white blood cell count, patients were divided into 3 groups: a significant portion (18.2%) had WBC > 25 × 10³/µL, half of the patients (50.4%) had WBC between 15 and 25 × 10³/µL, while a small percentage (8%) had a low WBC count < 4.5 × 10³/µL.

A prospective, observational, multicenter study showed that a substantial portion of patients (30.43%) had low albumin levels (< 3.0 U/L). Additionally, 19.99% had elevated alkaline phosphatase levels (> 125 U/L), with 16.45% showing elevated blood urea nitrogen levels (> 40 mg/dL) and 36.63% having elevated creatinine levels (> 1.2 mg/dL). Approximately 18.58% of the patients had an international normalized ratio (INR) greater than 1.5, and 15.97% had low platelet counts (< 150 × 10³/µL). Elevated SGOT levels (> 40 U/L) were found in 24.86% of the patients. Sodium levels above 145 mg/dL were less common, observed in 5.11% of patients. White blood cell (WBC) counts varied among patients, with counts < 4.5 × 10³/µL, > 15 and ≤ 25 × 10³/µL, and > 25 × 10³/µL representing 7.28%, 24.45%, and 8.66%, respectively [18].

Regarding ESS categories, the largest proportion of patients in this study (43.1%) fell into the high score category, while the Intermediate Score (4–11) category was the largest for both Greek patients (76%) and U.S. patients (63%) in a study conducted by Christou et al. [10].

Regarding postoperative complications, this study’s results showed that pneumonia was the most common complication, affecting nearly 60% of patients. Over half of the patients (52.6%) experienced superficial surgical site infections, and sepsis affected over a third of the patients (37.2%). More than a quarter (27%) of the patients required ventilator support for more than 48 h, and the same percentage experienced septic shock.

These results were higher than those of Han et al. [19], who found that 22% of patients experienced one or more postoperative infections, with the most common being surgical site infections (9%), pneumonia (5.7%), and sepsis/septic shock (12.2%) [19].

The complication rates were also greater than the findings of Alburakan et al. [12], who reported a sepsis rate of 5.5%, an organ/space surgical site infection rate of 3.7%, and a cardiac arrest rate of 2.2% among their patients [12].

The current study reported a mortality rate of nearly 28%. These results were higher than those reported by Alburakan et al. [12] and AlSowaiegh et al. [18], whose 30-day mortality rates were 2.2% and 14.8%, respectively. The high mortality rate in this study may be attributed to several factors, including the mean patient age of 48.66 ± 19.37 years, the high percentage of patients with comorbidities, and the high rate of complications observed, with pneumonia (affecting nearly 60%) and superficial surgical site infections (52.6%) being the most common.

The present study results showed a mean ESS of 12.55 ± 8.87, with minimum, median, and maximum values of 1, 8, and 28, respectively. These ESS values were higher than those reported in the study by AlSowaiegh et al. [18].

The ESS has been validated as an outcome prediction score in diverse populations through studies conducted in numerous countries, including the USA, the UK [9], Greece [10], Jordan [11], and Saudi Arabia [12].

The results of this study indicated that the ESS demonstrated excellent predictive ability for mortality, with an area under the curve (AUC) of 0.946, a sensitivity of 93.3%, and a specificity of 71%.

The association of ESS with 30-day mortality in Greek and U.S. patient groups was deemed satisfactory in the Greek cohort (c-statistic = 0.79 [95% CI: 0.67–0.90]) and excellent for 30-day mortality in the U.S. population (c-statistic = 0.83 [95% CI: 0.74–0.92]). For Greek patients, the mortality rates associated with ESS levels of 1, 6, 9, and 15 progressively escalated from 0 to 20%, 50%, and 100%, respectively [10].

Regarding ICU admission, the results of this study demonstrated that the ESS was highly effective at predicting the need for ICU admission, with an AUC of 0.950, a sensitivity of 91.4%, and a specificity of 60.9%.

Regarding postoperative complications, the results showed that the AUC of 0.839 was good, suggesting that the ESS had a strong predictive ability for postoperative complications. At a cutoff of 6, the sensitivity and specificity were 81.7% and 55.3%, respectively.

An observational study conducted in the Department of Surgery at Maulana Azad Medical College and its affiliated Lok Nayak Hospital in New Delhi reported c-statistics of 0.84 and 0.879 for the ESS in predicting postoperative morbidity and ICU length, respectively. A cutoff score of 7 was shown to be statistically significant for both ICU length (*p*-value = 0.00018) and postoperative problems (*p*-value = 0.04) [20].

A separate study determined that the ESS exhibited a strong correlation with mortality (c-statistic = 0.94) and the necessity for postoperative ICU care (c-statistic = 0.91), as well as a moderate correlation with morbidity (c-statistic = 0.77) [17].

## Conclusion

The ESS demonstrates its reliability as a predictor of 30-day mortality, surgical complications, and ICU admission within the Egyptian patient population. The ESS serves as a valuable tool for bedside perioperative counseling and for the triage of patients requiring ICU admission.

### Limitation of study

The primary limitation of this study is the follow-up period of 30 days. While this timeframe is valuable for assessing short-term outcomes, it may not fully capture the long-term performance or clinical utility of the scoring system. Future studies with extended follow-up periods are recommended to address this limitation. Additionally, the study was conducted without the use of a centralized or electronic database. Data were collected manually from patient records at two tertiary hospitals.

### Recommendation

This study supports the validity of the Emergency Surgery Score (ESS) in predicting 30-day postoperative outcomes in Egyptian patients undergoing emergency laparotomy. Given its simplicity, objectivity, and applicability across different populations, the integration of ESS into the routine preoperative assessment workflow in emergency surgical settings is recommended.

## Data Availability

No datasets were generated or analysed during the current study.
